# Case report: Psoriasiform eczema with immune-mediated comorbidities treated with upadacitinib

**DOI:** 10.3389/fimmu.2024.1432233

**Published:** 2024-08-05

**Authors:** Ilaria Salvi, Aurora Parodi, Emanuele Cozzani, Martina Burlando

**Affiliations:** ^1^ Department of Dermatology, Dipartimento di scienze della salute - DISSAL, University of Genoa, Genova, Italy; ^2^ IRCCS Ospedale Policlinico San Martino, Genova, Italy

**Keywords:** psoriasis; eczema, psoriasiform eczema, upadacitinib, dermatology, JAK inhibitor

## Abstract

Immune-mediated comorbidities in patients with psoriasiform eczema are common. It can be challenging to manage multiple immune-mediated diseases, especially considering that biologic treatments are prone to causing paradoxical effects. The aim of this retrospective observational case series was to describe the course of both psoriasiform eczema and immune-mediated comorbidities in five patients treated with upadacitinib for psoriasiform dermatitis. Five patients, all male, were included. All the patients suffered from psoriasiform eczema. Moreover, two of the patients suffered from alopecia areata, two from vitiligo, one from ulcerative colitis and one from hidradenitis suppurativa. In all cases, the treatment with upadacitinib was rapidly effective on the eczema. The effectiveness on alopecia areata was good in both cases, while the results on vitiligo were only partial. The only case of ulcerative colitis achieved complete remission, while the case of hidradenitis suppurativa experience partial improvement. In conclusion, upadacitinib was effective in treating not only psoriasiform eczema, but also several immune mediated comorbidities. Additional studies are necessary to determine the efficacy of upadacitinib in alopecia areata, vitiligo and hidradenitis suppurativa.

## 1 Introduction

The JAK-STAT pathway plays a vital role in various cellular processes. It is responsible for the expression of inflammatory mediators that are involved in many immune-mediated diseases (1). Cytokines binding to their receptor cause a rearrangement of the receptor subunits, which enables JAK activation by transphosphorylation. Activated JAKs phosphorylate the receptors, allowing STATs to bind to the receptor and become phosphorylated. The phosphorylated STATs form homo‐ or heterodimers and translocate into the nucleus where they bind their respective promoter elements, regulating the transcription of target genes. JAK inhibitors are small molecules aimed at modulating the JAK-STAT pathway, binding one or more members of the JAK family (JAK1, JAK2, JAK3, and TYK2) (1). Upadacitinib is an oral selective JAK-1 inhibitor, which functions as an adenosine triphosphate (ATP)‐competitive JAK inhibitor, competing with ATP and blocking nucleotide binding to inhibit kinase activity and the phosphorylation of downstream effectors, thus preventing the formation of STAT dimers, their translocation to the nucleus and promoter binding (1). Upadacitinib is effective in the treatment of rheumatoid arthritis, psoriatic arthritis, non-radiographic axial spondyloarthritis, ankylosing spondylitis, atopic dermatitis (AD), ulcerative colitis, and Crohn’s disease (2). For atopic dermatitis, a 15 mg or 30 mg once-daily administration is recommended. Phase 3 studies are currently underway to test the efficacy of upadacitinib in the treatment of dermatologic conditions such as hidradenitis suppurativa (HS) (2), vitiligo (3), and alopecia areata (4) (AA). The effectiveness of upadacitinib in these conditions has been demonstrated in phase 2 and real-life studies (5–7). Furthermore, various studies have shown that upadacitinib is effective in treating psoriasiform dermatitis (8, 9), a condition that shares features of both psoriasis and eczema which is becoming increasingly common, and is often observed in patients undergoing biological therapy for psoriasis or atopic dermatitis (10). Despite their efficacy in several diseases, JAK inhibitors have raised safety issues, having been associated to increased risk of tromboembolic events, infections and malignancies. However, it can be argued that the different selectivity of various JAK inhibitors could deeply influence both the spectrum of efficacy and the safety of each drug. In particular, selective JAK-1 inhibitors, such as upadacitinib, are expected to be less prone to cause the off-target effects of JAK inhibitors (11). This has been confirmed by real-life studies, that showed a low incidence of serious adverse events in patients with atopic dermatitis treated with upadacitinib (12).

We present a report on five cases of psoriasiform eczema associated with other immune-mediated diseases. In these cases, upadacitinib not only effectively treated the condition for which it was prescribed but also had varying degrees of effectiveness in treating the associated diseases.

## 2 Case presentation

**Figure 1:**
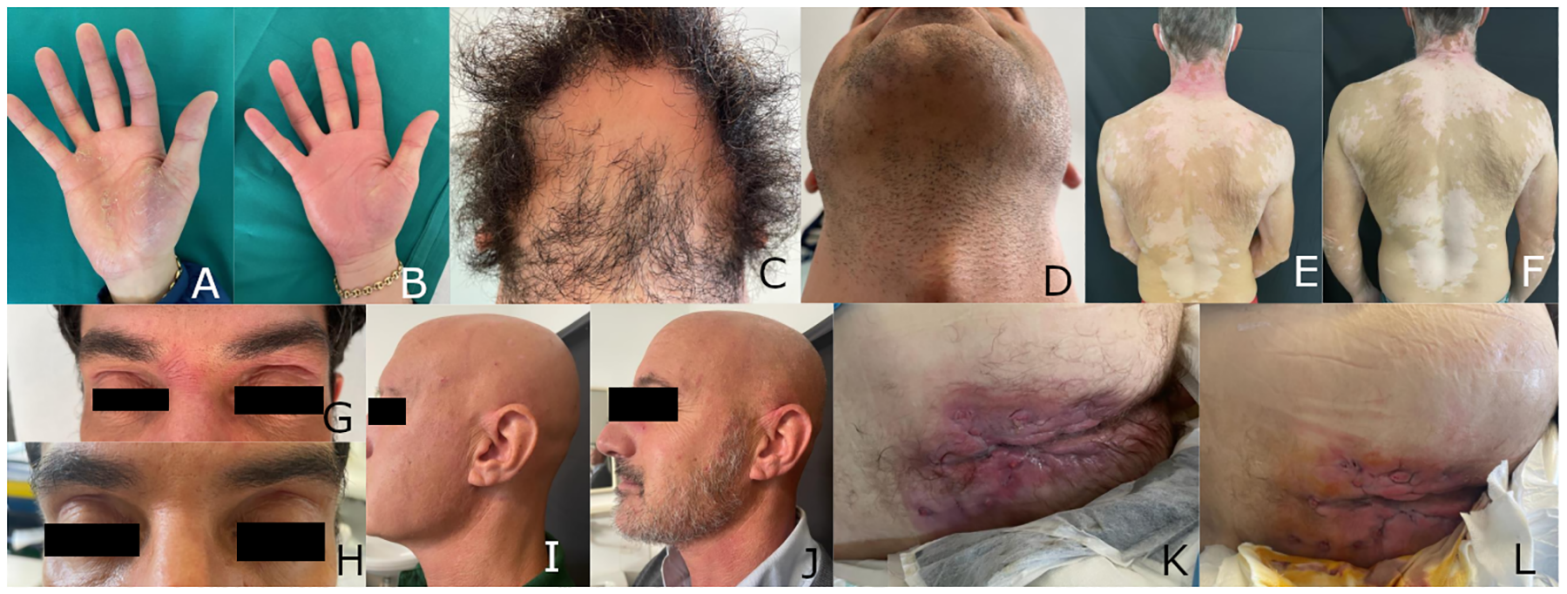
Patient 1: Psoriasiform eczema of the hand before **(A)** and three weeks after starting treatment with upadacitinib **(B)**; beard alopecia areata before **(C)** and three weeks after starting treatment with upadacitinib **(D)**. Patient 2: psoriasiform eczema and universal vitiligo before **(E)** and one month after starting treatment with upadacitinib **(F)**. Patient 3: psoriasiform eczema before **(G)** and four months after starting treatment with upadacitinib **(H)**. Patient 4: alopecia areata before **(I)** and four months after starting treatment with upadacitinib **(J)**. Patient 5: hidradenitis suppurativa before **(K)** and four weeks after starting treatment with upadacitinib **(L)**.

Patient 1, a 36-year-old man, had been suffering from palmoplantar psoriasis for a decade. He was treated with topical corticosteroids and later with acitretin, with little improvement. Upon examination, the patient’s condition appeared to be more like psoriasiform eczema (**Figure 1A**), which was confirmed by histopathology. The patient also had alopecia areata of the beard(**Figure 1C**), for which he had received topical corticosteroids treatment for years, with partial improvement.

The patient was prescribed upadacitinib at a daily dose of 30 mg. After only three weeks, he showed a marked improvement in the hand dermatitis (**Figure 1B**) and hair regrowth (**Figure 1D**). After six months, the eczema had resolved and beard alopecia had also almost disappeared. The patient was then switched to a lower dose of upadacitinib (15 mg), which he has been taking for over one year, with persistent clinical remission and no adverse events.

Patient 2, a 45-year-old male, had been suffering from atopic dermatitis since the age of 6, treated with topical steroids and, occasionally, oral steroids. At the age of 30, he was diagnosed with Hashimoto’s thyroiditis and started treatment with levothyroxine. A few years later, he developed vitiligo, which eventually spread to the entire body. At the age of 40, he reported the appearance of psoriasiform eczema at the elbows and knees. He was treated with cyclosporine first, interrupted for hypertension and abdominal pain, then methotrexate. Both drugs were only partially effective on the eczema and totally ineffective on vitiligo (**Figure 1E**).

In March 2023, it was decided to prescribe upadacitinib at a dose of 30 mg daily. After 4 months, there was a complete clearance of both psoriasis and atopic dermatitis. The eczema responded faster, with complete resolution in one month. After 8 months, scattered islands of pigmentation were noticed all over the body (**Figure 1F**). Therefore, it was decided to continue upadacitinib at a dose of 30 mg daily. To date, the patient has not experienced any adverse events.

Patient 3, a 35-year-old male, had been dealing with atopic dermatitis since the age of 6 months, initially treated with topical steroids. At the age of 12, he was diagnosed with ulcerative colitis and was prescribed mesalazine. In July 2021, his atopic dermatitis worsened, and he was prescribed dupilumab 300 mg every 2 weeks, with significant improvement. Unfortunately, the inflammatory bowel disease worsened, and, in July 2022 the patient was also prescribed ustekinumab 90 mg every 2 months, which resulted in partial clinical improvement, with a reduction of the Mayo Endoscopic score (13) from 2 to 1.

In February 2023, the patient developed severe conjunctivitis and psoriasiform dermatitis, which led to the discontinuation of dupilumab. No specific treatment for the dermatitis was prescribed as ustekinumab was also expected to control it. However, after three months, the dermatitis worsened (**Figure 1G**), and, in agreement with the gastroenterologist, ustekinumab was discontinued and upadacitinib was prescribed at a dose of 45 mg daily.

After four weeks of upadacitinib treatment, the patient’s dermatitis started to improve. After four months, the dermatitis resolved completely (**Figure 1H**), and the ulcerative colitis improved, with a reduction of the Mayo Endoscopic score (14) from 1 to 0.

Patient 4, a 46-year-old male, had been suffering from non-segmental vitiligo for 20 years and alopecia areata for 5 years. Initially, the alopecia was limited to a few patches in the nuchal region, but it eventually spread to the entire body, resulting in universal alopecia.

After unsuccessful cycles of treatment with topical steroids, the patient was treated with cyclosporine at a dose of 250 mg per day, but the treatment was discontinued after 6 months due to severe epigastralgia. The use of cyclosporine had only partially been effective on alopecia (**Figure 1I**), while the vitiligo remained unchanged.

The patient developed diffuse histopathologically-proven psoriasiform eczema of the upper extremities after discontinuing cyclosporine. Upadacitinib was prescribed at a dose of 15 mg per day, which resulted in the complete resolution of eczema and eyelash growth within four weeks. After four months, scalp hair also reappeared (**Figure 1J**) and pigmentation patches appeared on the trunk. After six more months, since the patients never experienced adverse events, the treatment with upadacitinib 15 mg is still ongoing, with slow improvement in both hair growth and pigmentation.

Patient 5, a 64-year-old male, had been suffering from Hurley III (14) hidradenitis suppurativa localized at the groin and intergluteal region and for over 10 years, treated with topical and systemic antibiotics, with limited efficacy. For the past 3 years, he had been experiencing chronic eczema on his hands. He underwent allergy tests which ruled out allergic contact dermatitis. The hand eczema was treated with topical and systemic steroids.

In July 2022, the patient began receiving adalimumab 40 mg injections weekly for his hidradenitis suppurativa, with partial benefit (from Hurley III to II) and a worsening of the eczema.

Six months later, methotrexate was prescribed at a dose of 15 mg weekly, followed by a folic acid tablet after 24h. Unfortunately, the addition of Methotrexate did not improve his condition (**Figure 1K**).

After six more months, it was decided to discontinue both adalimumab and methotrexate and to prescribe upadacitinib (30 mg daily). After four weeks, the patient’s hands and body eczema resolved and the hidradenitis suppurativa slightly improved (**Figure 1L**). To date, the eczema is in remission, and the hidradenitis suppurativa is slowly improving. The patient has experienced no adverse effects.

## 3 Discussion

The understanding of immune-mediated skin diseases has significantly deepened over the years, leading to the development of more effective therapeutic options. Monoclonal antibodies targeting the interleukin (IL)-4/IL-13 and the IL-17/IL-23 axes are available for the treatment of AD and psoriasis, respectively. However, these biologics may cause immunophenotypic cross-switching, and exacerbate or induce paradoxical reactions (15). For example, it has been described that treatment of AD with dupilumab and tralokinumab can lead to the onset of psoriasiform paradoxical reactions (16). Moreover, cases of patients with psoriasis or other chronic inflammatory systemic diseases treated with IL-17A inhibitors, IL-12/23 inhibitors, IL-23 inhibitors or TNF-α inhibitors developing eczematous paradoxical reactions, as well as paradoxical psoriasis, have been reported (15, 17). Since monotherapy does not always provide complete control of the disease and the possible associated comorbidities (18), in selected cases, dermatologists may take into consideration combining two biologic drugs. This has been reported both in psoriasis and in AD (17, 19), however, the safety and cost associated with the use of multiple therapies is a concern in clinical practice.

JAK inhibitors, including upadacitinib, may be considered the most suitable strategy for patients with multiple immune-mediated diseases (7, 8), with concomitant AD and psoriasis (10), as well in patients developing paradoxical psoriasis or eczema (16).

Moreover, since dysregulation of the JAK-STAT pathway is implicated in numerous chronic inflammatory skin conditions, it is not surprising that mounting evidence supports the efficacy of JAK inhibitors in other diseases, such as alopecia areata, hidradenitis suppurativa and vitiligo. In vitiligo, for example, IFN-γ produced by CD8+ T cells activates JAK1 and JAK2, leading to further recruitment of CD8+ T cells (20). IFNγ-mediated STAT1 activation is also implicated in the pathogenesis of hidradenitis suppurativa (21) and alopecia areata (22).

This article discusses five cases of patients who suffer from immune-mediated diseases, including psoriasiform eczema, treated with upadacitinib. The treatment was effective not only on the underlying pathology, but also the associated immune-mediated conditions.

Of note, the drug has different rates of action for different conditions, which are reported in **Table 1**. It appears to work quickly on psoriasiform eczema and alopecia, especially of the eyelashes, while it works more slowly on scalp and beard issues. For vitiligo, the first results are observed after 4 months. As for hidradenitis suppurativa, we only noticed slight improvement, which could signify a slower action in this disease.

**Table 1:** Results of upadacitinib treatment on immune-mediated comorbidities.

Patient ID	Gender	Age	Comorbidity	Site of comorbidity	Upadacitinib dosage (mg)	Time to effectiveness on psoriasiform eczema (weeks)	Result on comorbidity	Time to effectiveness on comorbidity (months)
**1**	M	36	Alopecia areata	Beard	30 for 6 months, later 15	3	Remission	6
**2**	M	45	Vitiligo	Universal	30	16	Partial improvement	8
**3**	M	35	Ulcerative colitis	Colon	45	16	Remission	4
**4**	M	46	Alopecia areata Vitiligo	Universal Trunk	15	4	Partial improvement Partial improvement	1 (lashes), 4 (scalp) 4
**5**	M	64	Hidradenitis suppurativa	Groin, intergluteal cleft	30	4	Slight improvement	1

Despite the safety concerns surrounding JAK inhibitors, upadacitinib, a selective JAK-1 inhibitor, has shown an acceptable safety profile in real-life studies (12). Indeed, none of the patients included in this case series presented adverse events during treatment with upadacitinib.

Further studies are certainly needed, but upadacitinib is proving to be an excellent therapeutic option for several dermatological diseases, even when they coexist in the same patient.

## Data availability statement

The original contributions presented in the study are included in the article/supplementary material. Further inquiries can be directed to the corresponding author.

## Ethics statement

Ethical approval was not required for the studies involving humans because ethical approval is not required for case reports. The studies were conducted in accordance with the local legislation and institutional requirements. The participants provided their written informed consent to participate in this study. Written informed consent was obtained from the individual(s) for the publication of any potentially identifiable images or data included in this article.

## Author contributions

IS: Conceptualization, Visualization, Writing – original draft, Writing – review & editing. AP: Methodology, Supervision, Visualization, Writing – review & editing. EC: Supervision, Visualization, Writing – review & editing. MB: Conceptualization, Data curation, Investigation, Methodology, Writing – review & editing.
